# A History of Chagas Disease Transmission, Control, and Re-Emergence in Peri-Rural La Joya, Peru

**DOI:** 10.1371/journal.pntd.0000970

**Published:** 2011-02-22

**Authors:** Stephen Delgado, Ricardo Castillo Neyra, Víctor R. Quispe Machaca, Jenny Ancca Juárez, Lily Chou Chu, Manuela Renee Verastegui, Giovanna M. Moscoso Apaza, César D. Bocángel, Aaron W. Tustin, Charles R. Sterling, Andrew C. Comrie, César Náquira, Juan G. Cornejo del Carpio, Robert H. Gilman, Caryn Bern, Michael Z. Levy

**Affiliations:** 1 School of Geography and Development, University of Arizona, Tucson, Arizona, United States of America; 2 Division of Epidemiology and Biostatistics, University of Arizona, Tucson, Arizona, United States of America; 3 Bloomberg School of Public Health, Johns Hopkins University, Baltimore, Maryland, United States of America; 4 Urbanización Ingeniería, Universidad Peruana Cayetano Heredia, Lima, Peru; 5 Vanderbilt University School of Medicine, Nashville, Tennessee, United States of America; 6 Department of Veterinary Science and Microbiology, University of Arizona, Tucson, Arizona, United States of America; 7 Gerencia Regional de Salud de Arequipa, Arequipa, Peru; 8 Division of Parasitic Diseases and Malaria, Center for Global Health, Centers for Disease Control and Prevention, Atlanta, Georgia, United States of America; 9 Department of Biostatistics and Epidemiology, University of Pennsylvania, Philadelphia, Pennsylvania, United States of America; Universidad de Buenos Aires, Argentina

## Abstract

**Background:**

The history of Chagas disease control in Peru and many other nations is marked by scattered and poorly documented vector control campaigns. The complexities of human migration and sporadic control campaigns complicate evaluation of the burden of Chagas disease and dynamics of *Trypanosoma cruzi* transmission.

**Methodology/Principal Findings:**

We conducted a cross-sectional serological and entomological study to evaluate temporal and spatial patterns of *T. cruzi* transmission in a peri-rural region of La Joya, Peru. We use a multivariate catalytic model and Bayesian methods to estimate incidence of infection over time and thereby elucidate the complex history of transmission in the area. Of 1,333 study participants, 101 (7.6%; 95% CI: 6.2–9.0%) were confirmed *T. cruzi* seropositive. Spatial clustering of parasitic infection was found in vector insects, but not in human cases. Expanded catalytic models suggest that transmission was interrupted in the study area in 1996 (95% credible interval: 1991–2000), with a resultant decline in the average annual incidence of infection from 0.9% (95% credible interval: 0.6–1.3%) to 0.1% (95% credible interval: 0.005–0.3%). Through a search of archival newspaper reports, we uncovered documentation of a 1995 vector control campaign, and thereby independently validated the model estimates.

**Conclusions/Significance:**

High levels of *T. cruzi* transmission had been ongoing in peri-rural La Joya prior to interruption of parasite transmission through a little-documented vector control campaign in 1995. Despite the efficacy of the 1995 control campaign, *T. cruzi* was rapidly reemerging in vector populations in La Joya, emphasizing the need for continuing surveillance and control at the rural-urban interface.

## Author Summary

The historically rural problem of Chagas disease is increasing in urban areas in Latin America. Peri-rural development may play a critical role in the urbanization of Chagas disease and other parasitic infections. We conducted a cross-sectional study in an urbanizing rural area in southern Peru, and we encountered a complex history of Chagas disease in this peri-rural environment. Specifically, we discovered: (1) long-standing parasite transmission leading to substantial burden of infection; (2) interruption in parasite transmission resulting from an undocumented insecticide application campaign; (3) relatively rapid re-emergence of parasite-infected vector insects resulting from an unsustained control campaign; (4) extensive migration among peri-rural inhabitants. Long-standing parasite infection in peri-rural areas with highly mobile populations provides a plausible mechanism for the expansion of parasite transmission to nearby urban centers. Lack of commitment to control campaigns in peri-rural areas may have unforeseen and undesired consequences for nearby urban centers. Novel methods and perspectives are needed to address the complexities of human migration and erratic interventions.

## Introduction

An estimated 8 million people in Latin America are infected by the protozoan parasite *Trypanosoma cruzi*, the causative agent of Chagas disease [1]. *Trypanosoma cruzi* is typically transmitted to humans and other mammals through contact with feces of an infected blood-feeding triatomine insect. The primary vector species in southern Peru is *Triatoma infestans*, which has adapted to live in and around human dwellings. Infection can also occur via congenital transmission, blood transfusion, or organ transplantation [2]. Infection is generally life-long and while most infected individuals remain asymptomatic, 20–30% progress over a period of decades to chronic clinical manifestations, including life-threatening cardiac and/or gastrointestinal disease [3]. As a result, Chagas disease is estimated to be responsible for the loss of 670,000 disability-adjusted life years (DALYs) and 14,000 human lives annually [4].

*Trypanosoma cruzi* transmission by *T. infestans* has been interrupted in several South American countries through household application of pyrethroid insecticides, but a comprehensive approach to vector control has only recently been instituted in southern Peru [1], [5]. Throughout Latin America, however, Chagas disease vector control is complicated by the processes of urbanization and migration [6], [7]. In recent decades in southern Peru, extensive urbanization has occurred at the periphery of cities as well as within previously rural areas [8]. New localities are typically established by rural migrants and share the trait of being situated – geographically as well as socio-culturally – at a rural-urban interface [9].

To improve understanding of *T. cruzi* transmission in the peri-rural context, we performed cross-sectional serological and entomological surveys in four contiguous localities located 30 km from the city of Arequipa. We evaluated spatial and temporal patterns of *T. cruzi* infection, utilizing a multivariate catalytic model [10] and Bayesian methods to estimate incidence of infection over time.

## Methods

### Ethics statement

The ethical review committees of the Johns Hopkins Bloomberg School of Public Health, the Universidad Peruana Cayetano Heredia, and the University of Pennsylvania approved the research protocol. The ethical review committee of the University of Arizona approved the usage of de-identified study data. All individuals ≥1 year old residing within the study area were invited to participate in the serological study. Signed informed consent was obtained prior to participation by adults and parents of participating children. Children also provided signed informed assent prior to participating. All households in the study area were invited to participate in the entomological study. Signed informed consent was obtained prior to participation by an adult resident of each household.

### Study area and population

**Figure 1:**
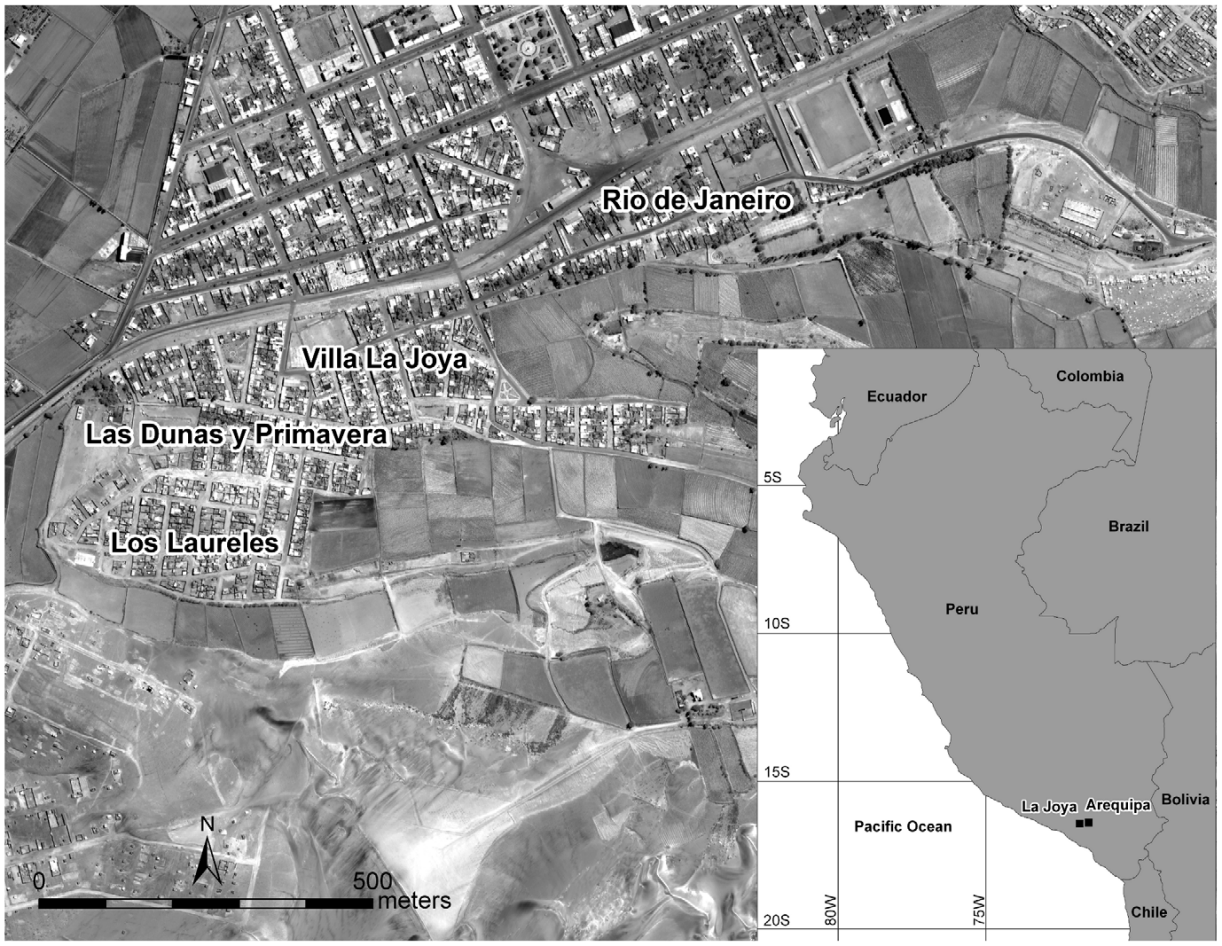
The La Joya, Peru, study area. The high-resolution satellite image (50-cm resolution panchromatic image acquired by WorldView-1 on 04 September 2008, eMap International) shows the peri-rural landscape of the La Joya study area. The inset map illustrates the proximate locations of the La Joya study site and the city of Arequipa.

The district of La Joya (population 24,192) is located approximately 30 km southwest of the city of Arequipa (population 864,250) and encompasses a mosaic of rural and peri-rural communities [8]. In this article, peri-rural refers to communities with high-density human habitation within an otherwise rural landscape, whereas peri-urban describes localities with high-density human habitation located at the periphery of an urban center [9]. The La Joya study area (Figure 1) was comprised of four contiguous peri-rural localities, with 2,251 persons living in 678 households within a 41-hectare area. From August through November 2008, we conducted human and entomological surveys. The human survey included a collection of demographic data and a detailed history of where each individual had lived from birth to the present time. The human survey and vector collections were timed to coincide with a household insecticide spray campaign conducted by the Arequipa Regional Office of Health. No documentation of any previous household insecticide campaign in the area was found in the Chagas Control Program records prior to the study.

### Serological and socio-demographic data collection

Detailed socio-demographic data, including a comprehensive migration history, were collected from each consenting study participant. Migration data were coded to indicate whether each individual lived inside or outside of the study area during each calendar year over the span of his or her lifetime. Five ml of venous blood was drawn from each study participant by trained medical personnel (3 ml for children younger than 5 years). Blood was maintained at 4°C and separated by centrifugation on the day of collection. Serum and cellular fractions were stored at −20°C until testing. Serum specimens were evaluated for antibodies to *T. cruzi* using a commercially available enzyme-linked immunosorbent assay (ELISA) (Chagatek, Laboratorio Lemos). The assay is reported to have 100% (95% CI: 82.9–100%) sensitivity; specificity estimates have varied from 87.3% (80.9–92.2%) to 98.1% (93.8–99.7%) [11]. All specimens with positive ELISA results and a 20% random sample of specimens with negative results were tested by the indirect immunofluorescent antibody test (IFA) using a titer of 1:32 as the positive cutoff [12]. Specimens with a positive result for both ELISA and IFA were considered confirmed positive for *T. cruzi* infection.

### Entomological and parasitological data collection

Immediately after application of the residual pyrethroid insecticide deltamethrin 5% Wettable Powder (K-Othrine, Bayer) at 25 mg a.i. per m^2^, two trained triatomine collectors systematically searched all domestic and peri-domestic areas, which included human and animal living spaces, for a total of one person-hour for each participating household [13]. Captured *T. infestans* were transported the same day to the Arequipa-based Universidad Peruana Cayetano Heredia laboratory. Insect life stage, sex (adults), and count were documented by site of collection. Gut contents of all insects (except first instars) were evaluated microscopically to determine *T. cruzi* infection status [14].

### Geographic data collection

Household geographic coordinates were collected using a global positioning system (GPS) unit (Juno ST, Trimble Navigation Limited). Geographic coordinates; household and individual participant codes; and diagnostic, socio-demographic, and entomological-parasitological data were maintained in a relational database management system and imported into a geographic information system (GIS) (ArcGIS 9.3.1, ESRI) for subsequent analysis and visualization.

### Statistical analysis

#### Univariate statistical analysis

Univariate statistical analyses were used to assess associations between participant *T. cruzi* serostatus and potential risk factors, which included socio-demographic characteristics and household entomological-parasitological conditions. The χ^2^ test was used for discrete covariates and the Wilcoxon rank-sum test for continuous covariates. Statistical tests were conducted using Stata 10 (StataCorp).

#### Spatial analysis

Using a GIS, household locations were mapped to visualize the spatial distributions of *T. cruzi*-seropositive, *T. infestans*-positive, and *T. infestans T. cruzi*-positive households. To preserve study participant confidentiality while maintaining actual and relative distance and orientation relationships, locations of all households were masked by displacing each point from its original location by a constant increment [15].

We employed K-function difference analysis to assess spatial clustering of households by serological, entomological, and entomological-parasitological status. The K-function, K(d), measures the total number of observations occurring within a given distance of any specified observation and describes the degree of spatial dependence of a set of observations [16]. Evaluation of the difference in K-functions for positive versus negative observations provides an estimation of the relative degree of clustering across specified spatial scales [17]. Specifically, we evaluated K-function differences for *T. cruzi*-seropositive versus seronegative, *T. infestans*-positive versus negative households, and *T. infestans T. cruzi*-positive versus negative households at spatial scales from 10 to 500 m in 10-m increments. Statistical significance of results was assessed using a 9999-iteration Monte Carlo random labeling simulation [18]. Spatial statistical analyses were conducted in R 2.9.2 [19].

#### Bayesian Markov Chain Monte Carlo regression analysis

We expanded upon the classic catalytic model [10] and utilized Bayesian Markov Chain Monte Carlo (MCMC) methods to estimate temporal and geographical parameters of individual *T. cruzi* infection risk. We evaluated three alternative independent models:

1. **Age-only model:** A basic catalytic model in which infection probability is modeled as a function of an individual's age and an intercept term:
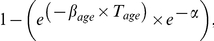
where 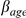 represents the average annual risk of infection per year of age; 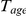 represents an individual's age in years.

2. **Migration-only model:** An expanded catalytic model in which infection probability is modeled as a function of an individual's years of residence inside and outside the study area and an intercept term:
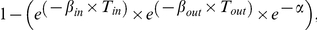
where 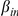 and 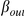 represent the average annual risk of infection per year of residence inside and outside the study area, respectively; 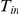 and 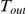 represent an individual's years of residence inside and outside the study area, respectively.

3. **Migration-transmission interruption model:** An expanded catalytic model in which infection probability is modeled as a function of an individual's years of residence inside and outside the study area, where residence in the study area is divided into time before and after a postulated insecticide application campaign, and an intercept term:
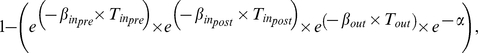
where 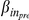, 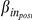, and 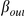 represent the average annual risk of infection per year of residence inside the study area before a postulated intervention campaign, after a postulated intervention campaign, and outside the study area, respectively; 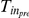, 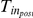, and 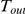 represent an individual's years of residence inside the study area before a postulated intervention campaign, after a postulated intervention campaign, and outside the study area, respectively. For all models, the intercept term, 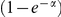, represents the probability of *T. cruzi* infection independent of time of exposure, including the probability of congenital transmission of *T. cruzi* infection. All β and α coefficients were assumed to be constant and independent of each other.

For each model, uniform prior distributions were specified for β coefficients (0–0.05), the 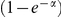 term (0–0.05), and the number of years since a postulated intervention campaign (0–50). Models were fit using a 200,000-iteration Metropolis-Hastings MCMC algorithm [20], with a 100,000-iteration burn-in and 100-fold thinning factor. Regression coefficients and 95% credible intervals were derived from posterior distributions. Model convergence was evaluated using the Gelman-Rubin statistic [21]. Goodness-of-fit of the three models was compared using the deviance information criterion (DIC) [22].

## Results

### Univariate statistical analysis results

Of 2,251 study area residents, 1,333 (59.2%) participated in the serological survey. Compared with study participants, non-participants were younger (mean age  = 22.6 versus 26.6 years; p<0.001) and were more likely to be male (57.1% versus 42.2%; p<0.001). One hundred forty-four participants had positive results by *T. cruzi* ELISA (10.8%; 95% CI: 9.1–12.5%), and 101 (7.6%; 95% CI: 6.2–9.0%) had confirmed positive results by IFA. Forty-three individuals had ELISA-positive/IFA-negative diagnostic results and were considered seronegative in subsequent analyses. Of 238 randomly selected ELISA-negative specimens, 226 were IFA-negative and 12 were IFA-positive. All were considered seronegative in subsequent analyses. ELISA-IFA inter-diagnostic concordance was evaluated using the kappa statistic: kappa  = 0.687 (95% CI: 0.610–0.764). All *T.cruzi* seropositive individuals were evaluated by Arequipa Regional Office of Health medical personnel and were offered treatment in accordance with the Ministry of Health of Peru guidelines.

Study participants contributed a total of 35,501 person-years at risk for *T. cruzi* infection, 19,514 (55.0%) of which were experienced inside the study area and 15,987 (45.0%) of which were experienced outside the study area. The prevalence of *T. cruzi* infection increased with increasing age, years of residence inside the study area, and years of residence outside the study area (Table 1). Of 513 study participants aged 18 years or younger, only five (1.0%; 95% CI: 0.1–1.8%) had confirmed positive serological results, and three of these were children of *T. cruzi* seropositive mothers. There were no significant associations between individuals' serostatus and the presence or density of *T. infestans* or *T. cruzi*-infected *T. infestans* in their households (Table 2).

**Table 1:** Associations between study participants' *T. cruzi* serostatus and their socio-demographic characteristics.

Socio-demographic characteristics	Population			p-value
	Total (N = 1,333)	*T. cruzi* seropositive (n = 101)	*T. cruzi* seronegative (n = 1,232)	
age^a^	24 (13–37)	40 (28–56)	23 (12–36)	<0.0001^b^
time of residence inside study area^a^	11 (5–21)	25 (16–35)	11 (5–19)	<0.0001^b^
time of residence outside study area^a^	8 (0–20)	15 (1–23)	7 (0–20)	0.0083^b^
sex	57.8% female (n = 770)	60.4% female (n = 61)	57.5% female (n = 709)	0.578^c^
	42.2% male (n = 563)	39.6% male (n = 40)	42.5% male (n = 523)	

a median (inter-quartile range) in years.

b Wilcoxon rank-sum test.

c Pearson χ^2^ test.

Note: Age data were missing for one individual, who was *T. cruzi* seronegative. Time of residence data were missing for seven individuals, all of whom were *T. cruzi* seronegative. These individuals were excluded from these analyses.

**Table 2:** Associations between study participants' *T. cruzi* serostatus and the entomological-parasitological conditions of their households.

Household entomological parasitological conditions	Total (N = 1,333)	*T. cruzi* seropositive (n = 101)	T. cruzi seronegative (n = 1,232)	p-value
*T. infestans*-positive	38.8% (n = 512)	37.6% (n = 38)	38.9% (n = 474)	0.803^b^
*T. infestans*-negative	61.2% (n = 808)	62.4% (n = 63)	61.1% (n = 745)	
*T. cruzi*-infected *T. infestans*-positive	10.2% (n = 134)	7.9% (n = 8)	10.3% (n = 126)	0.440^b^
*T. cruzi*-infected *T. infestans*-negative	89.8% (n = 1,186)	92.1% (n = 93)	89.7% (n = 1,093)	
*T. infestans* per household^a^	0 (0–3)	0 (0–3)	0 (0–3)	0.925^c^
*T. cruzi*-infected *T. infestans* per household^a^	0 (0-0)	0 (0-0)	0 (0-0)	0.457^c^

a median (inter-quartile range) in number of *T. infestans.*

b Pearson χ^2^ test.

c Wilcoxon rank-sum test.

Note: Thirteen individuals, all of whom were *T. cruzi* seronegative, were missing data regarding household entomological parasitological conditions. These individuals were excluded from these analyses.

### Spatial analysis

**Figure 2:**
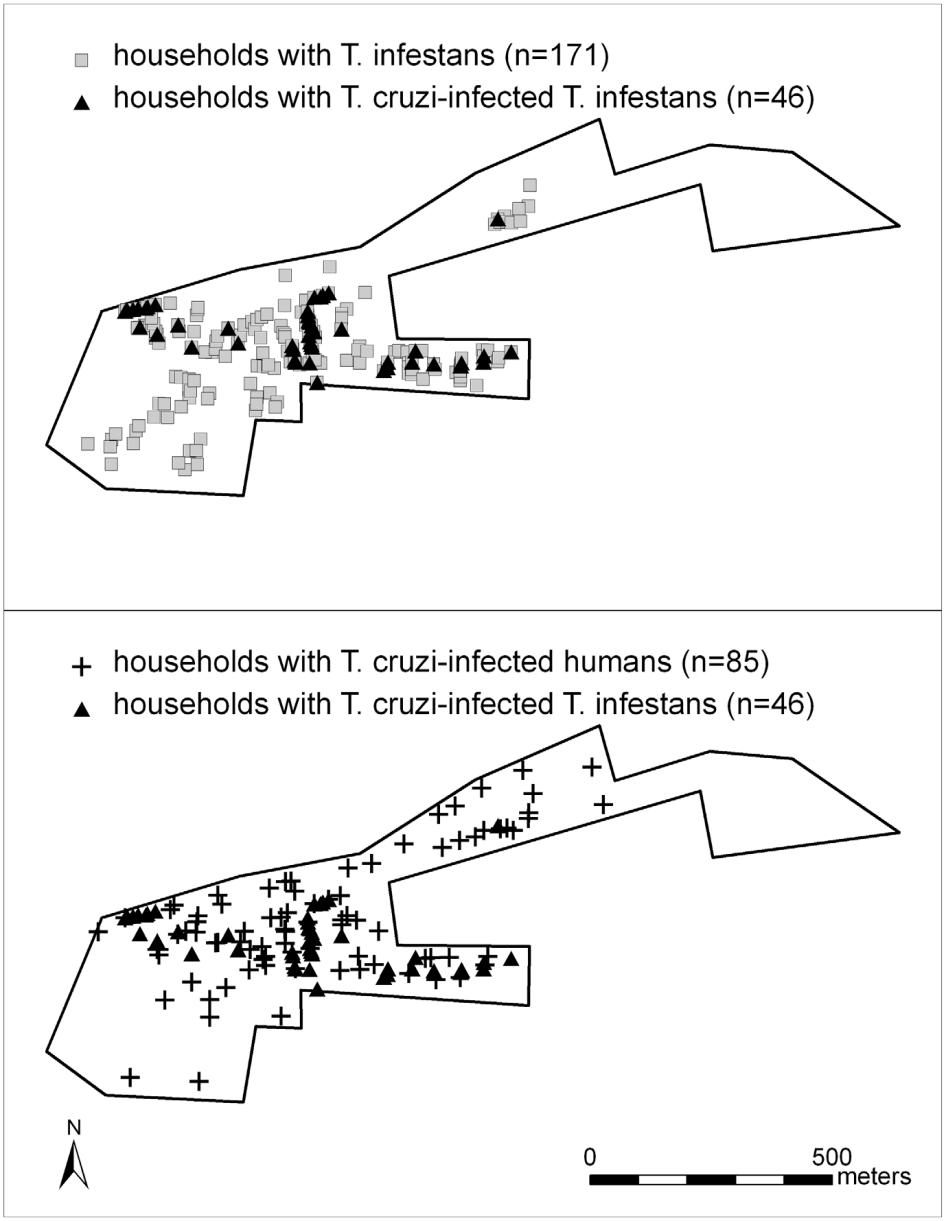
Spatial patterns of *T. cruzi* infection in *T. infestans* and humans. Comparisons of spatial distributions of households with *T. infestans* versus households with *T. cruzi*-infected insects (top panel); and households with *T. cruzi*-seropositive humans versus households with *T. cruzi*-infected insects (bottom panel). To preserve study participant confidentiality while maintaining actual and relative distance and orientation relationships, locations of all households were masked by displacing each point from its original location by a constant increment.

Of 678 study area households, 405 (59.7%) had one or more residents who participated in the serological survey. Eighty-five (21.0%; 95% CI: 17.0–25.0%) households surveyed contained at least one person with *T. cruzi* infection. Six hundred twenty-three (91.9%) households participated in the entomological survey, and *T. infestans* were collected in 171 (27.4%; 95% CI: 23.9–31.0%) households surveyed. Of the 170 infested households with vectors examined, 46 (27.1%; 95% CI: 20.3–33.8%) contained *T. cruzi*-positive vectors (Figure 2).

**Figure 3:**
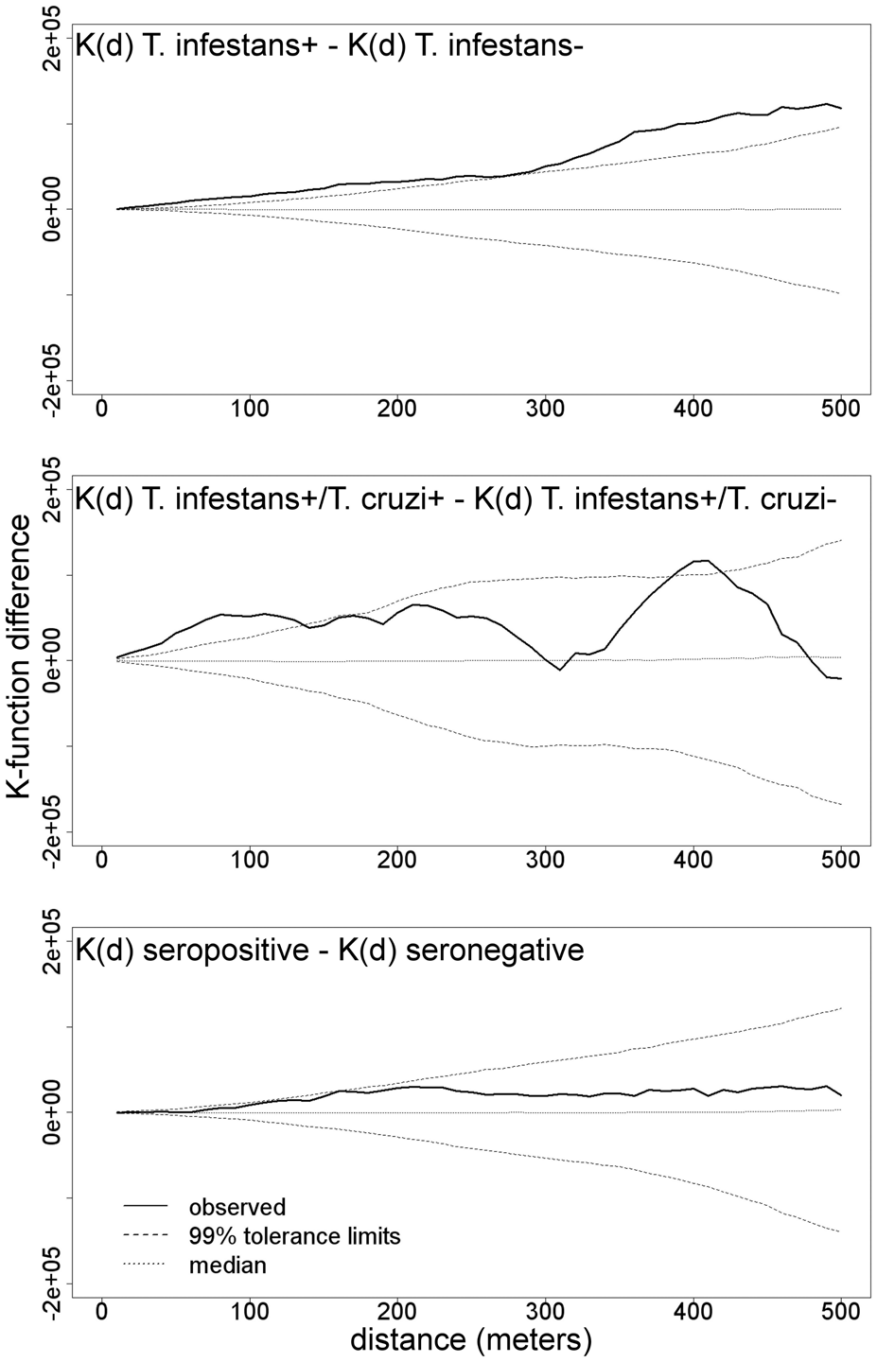
Spatial clustering analysis of *T. infestans*, *T. cruzi*-infected *T. infestans*, and *T. cruzi*-seropositive humans. K-function difference analyses, where observed values above 99% tolerance limits indicate statistically significant spatial clustering at a given spatial scale. Clustering of households with *T. infestans* was statistically significant at all spatial scales from 10 to 500 m (top panel), while clustering of households with *T. cruzi*-infected vectors was statistically significant only at spatial scales from 10 to 130 m and 390 to 410 m (middle panel). Clustering of households with *T. cruzi*-seropositive humans was not statistically significant at any spatial scale from 10 to 500 m (bottom panel).

K-function difference analysis demonstrated statistically significant spatial clustering of vector-infested households at spatial scales from 10 to 500 m. Similar analysis found significant spatial clustering of households with *T. cruzi*-infected vectors at spatial scales from 10 to 130 m and 390 to 410 m. No significant clustering was found at any spatial scale evaluated for households containing *T. cruzi*-infected humans (Figure 3).

### Bayesian Markov Chain Monte Carlo regression analysis results

**Figure 4:**
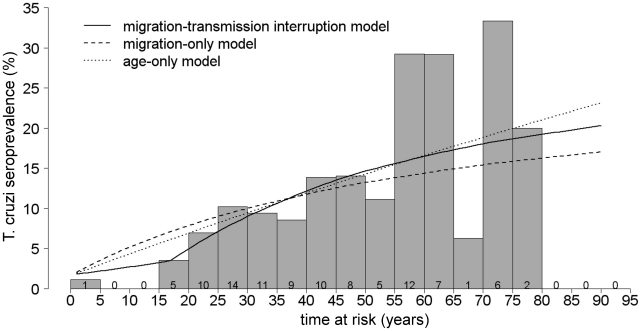
A comparison of catalytic model predictions and actual age-specific *T. cruzi* seroprevalence. Curves show the predicted prevalence of *T. cruzi* infection versus time at risk for each of the three regression models evaluated. A histogram of seroprevalence by five-year age category is provided for comparison of model and empirical results. The value inside each bar represents the number of *T. cruzi*-seropositive individuals in each 5-year age category.

The migration-transmission interruption model provided the best fit to the seroprevalence data (Table 3, Figure 4). This model indicated that transmission was interrupted around 1996, approximately 12 years prior to our study. Based on model estimates, the mean annual incidence of *T. cruzi* infection in the study area was approximately 1% prior to transmission interruption and fell to 0.1% from then onward. The mean incidence per year of residence outside the study area was approximately 0.1%. The intercept value 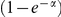 was estimated to equal 0.5%. This value represents the probability of *T. cruzi* infection independent of time of exposure, including the probability of congenital infection.

**Table 3:** Catalytic regression models, coefficient estimates, 95% credible intervals, and deviance information criterion (DIC) values.

Model parameters	Model specifications		
	age-only model^a^	migration-only model^b^	migration-transmission interruption model^c^
***I*** ** = years since intervention campaign**	---	---	12 (8–17)
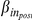 ** = risk of infection per year of residence inside the study area after the intervention campaign**	---	---	9.8×10^−4^ (5.5×10^−5^−2.7×10^−3^)
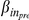 ** = risk of infection per year of residence inside the study area before the intervention campaign**	---	---	9.2×10^−3^ (6.2×10^−3^−1.3×10^−2^)
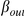 ** = risk of infection per year of residence outside the study area**	---	1.0×10^−3^ (2.4×10^−4^−2.0×10^−3^)	1.5×10^−3^ (6.0×10^−4^−2.4×10^−3^)
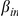 ** = risk of infection per year of residence inside the study area**	---	4.7×10^−3^ (3.4×10^−3^−5.9×10^−3^)	---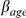 ** = risk of infection per year of age**	2.9×10^−3^ (2.4×10^−3^−3.6×10^−3^)	---	---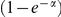 ** = probability of infection independent of time of exposure**	3.7×10^−3^ (2.1×10^−4^−1.2×10^−2^)	3.1×10^−3^ (1.0×10^−4^−1.0×10^−2^)	4.5×10^−3^ (1.9×10^−4^−1.3×10^−2^)
**deviance information criterion (DIC)**	644.0	629.6	614.4

a
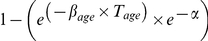

b
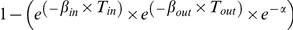

c
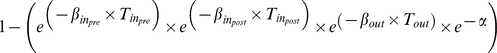

Note: Age data were missing for one individual, who was *T. cruzi* seronegative. Time of residence data were missing for seven individuals, all of whom were *T. cruzi* seronegative. These individuals were excluded from these analyses.

**Figure 5:**
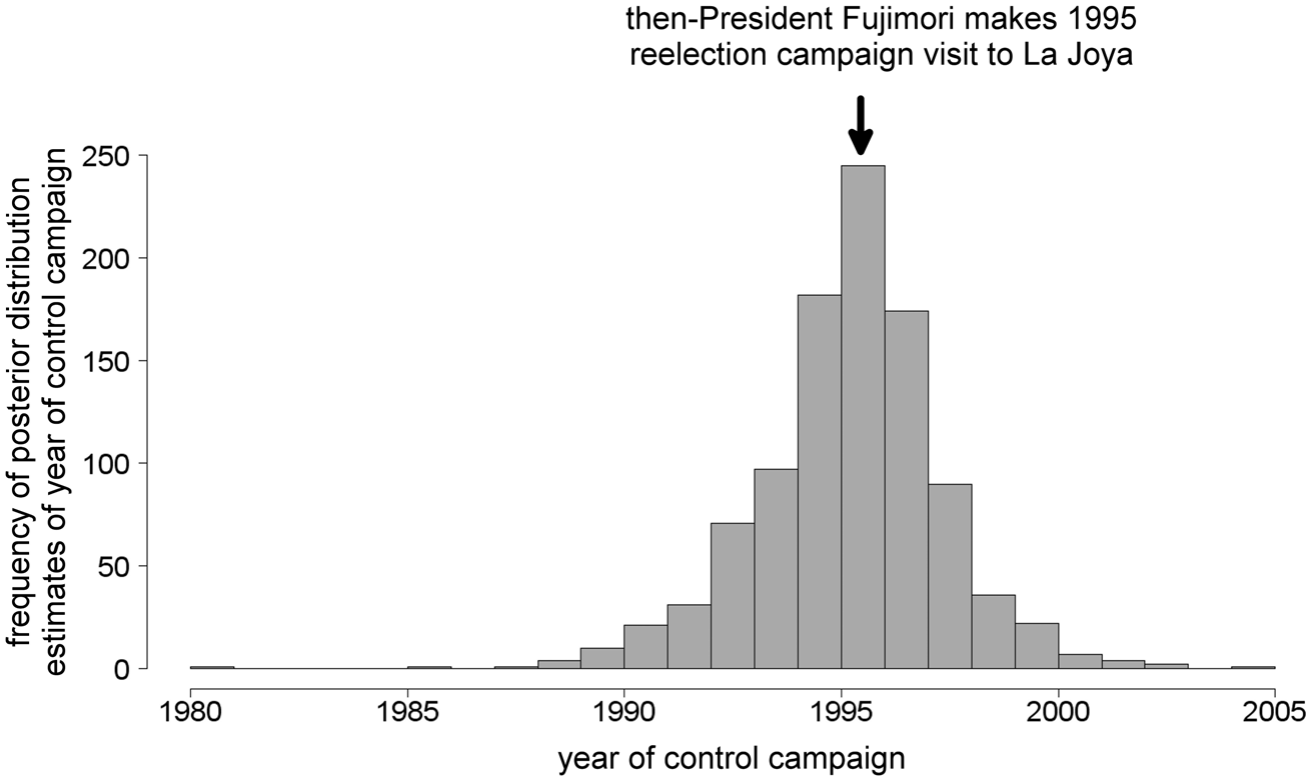
A comparison of catalytic model prediction and historical data. An expanded catalytic model and a Bayesian MCMC algorithm were used to estimate the year of an insecticide intervention in La Joya. The frequency histogram shows the distribution of the 1,000 estimates generated by the model. Model prediction of the year of the intervention was confirmed by historical documentation of a campaign authorized by then-President Fujimori as part of his reelection campaign.

Based on model results, we interviewed Ministry of Health personnel who had been working since at least the early 1990s. Several officials recounted that then-President Alberto Fujimori visited La Joya in 1995 as part of his re-election campaign and authorized financing for an insecticide spray campaign in response to local political demands. Ministry field personnel confirmed that a single, district-wide application of the pyrethroid insecticide 10% lambda-cyhalothrin at 25 mg a.i. per m^2^ was implemented in May to October 1995. We subsequently searched local newspaper archives and encountered an article documenting the control campaign [23]. Data obtained from personal interviews and newspaper records strongly support model estimation of transmission interruption (Figure 5).

## Discussion

The transmission of *Trypanosoma cruzi* by *Triatoma infestans* can be interrupted through coordinated and sustained vector control efforts, as has been demonstrated in Brazil, Chile, and Uruguay [24]. The history of Chagas disease control in Peru and many other nations, however, is marked by sporadic and poorly documented vector control campaigns [25]. These campaigns, although ephemeral, can affect the patterns of infection in a population. Through a catalytic model we were able to reconstruct the history of *T. cruzi* transmission in the peri-rural community of La Joya, Peru, and in the process describe the effect of a nearly forgotten vector control campaign.

The classic catalytic model has been utilized widely to retrospectively estimate incidence from age-prevalence data for a variety of lifelong infections [26]–[28], including *T. cruzi* infection [29]–[31]. The basic model assumes that incidence of infection is constant with time and age; we show here that the model can be easily expanded to accurately describe a complex history of transmission and control, even in a peri-rural community with a highly mobile population. We found that incidence of *T. cruzi* infection was high prior to a vector control campaign in 1995. This control campaign successfully disrupted transmission of *T. cruzi.* Our estimates from the expanded catalytic model show prevalence in the study population increasing from 5% in twenty year olds, up to 10% among thirty year olds and 20% among those over sixty. Little of the prevalence could be attributed to infection outside of the study area.

Peri-rural and peri-urban places play a crucial role in contemporary dynamics of migration and urbanization across Latin America [9]. In turn, migration and urbanization are changing the geography and epidemiology of many parasitic infections, including Chagas disease [6], [7]. The incursion of *T. infestans* and *T. cruzi* into nearby urban and peri-urban Arequipa [32], [33] may have its source in peri-rural communities like La Joya. Inhabitants of peri-urban Arequipa typically migrate to nearby rural and peri-rural localities in search of seasonal agricultural employment [34]. The cycle of seasonal migration provides a plausible means for the rural-to-urban transportation of vector and parasite. The timing, at least, is suggestive. *Triatoma infestans* emerged in peri-urban communities of Arequipa in the 1980s and 1990s [34]. Our analysis here shows that transmission was very common in La Joya in the 80s and midway through the 90s. Molecular studies [35] of the vector and parasite could provide a definitive answer to the question of provenance of Chagas disease in and around the city of Arequipa.

The intervention that nearly eliminated *T. cruzi* transmission in the study communities consisted of a single application of pyrethroid insecticide. The intervention effectively stopped *T. cruzi* transmission for many years. However, as is often the case, over time *T. infestans* and *T. cruzi* reemerged in the end [25]. The spatial patterns we observed in La Joya are precisely those to be expected following a reasonably effective intervention campaign. A large number of older individuals were infected, and they presumably were infected prior to the intervention campaign by vector populations that were spatially diverse. The vectors we observed during our study were clustered, suggesting that the vector population is so young that it was still in the process of re-dispersing through the community. Households with vectors carrying the parasite were even more spatially clustered, suggesting that the re-dispersal of *T. infestans* has outpaced that of *T. cruzi*. We found very low prevalence of infection in children, and some of the few children diagnosed may well have been infected congenitally. The resurgent *T. cruzi* transmission clearly had not yet caused many infections in humans. However, had the parasite been allowed to continue to spread there is little doubt that it would have resulted in significant disease in the human population.

The contrasting presence of *T. cruzi* in the vector population and absence of new human infections suggest that parasite reemergence was limited to animal reservoirs. There are diverse and dense domestic animal populations in peri-rural communities of Peru, and La Joya is no exception. The guinea pig is particularly common, and is a highly competent reservoir for *T. cruzi*. We speculate that the presence of high densities of guinea pigs has allowed for reemergence of *T. cruzi*.

At least three important study limitations merit mention. First, *T. cruzi* diagnostics lack a gold standard, and are especially difficult to interpret in Arequipa [36]. We considered only confirmed (ELISA-positive/IFA-positive) cases of infection; spatial analyses elsewhere in Arequipa suggests that ELISA-positive/IFA-negative results likely represent true positives [37]. Accordingly, our estimates of seroprevalence and incidence in the study population may be lower than the true values. Second, non-participants were on average slightly younger than participants, which might lead to a slight underestimate of seroprevalence in the population, though this would not affect the incidence estimates from the catalytic model. Third, we were unable to consider a more detailed model of geographic variability in risk of *T. cruzi* infection. This limitation resulted from the vast variety of study participants' migration histories, which included many small towns that we could not locate.

We attempted to gather information on the previous insecticide control activities in the study site prior to conducting our study. In-depth interviews conducted with community members failed to elicit recall of the 1995 insecticide application campaign [34]. Insecticide application was simply not a memorable event in the lives of community members. We may have lacked some due diligence in examining files in the health post; these were not made readily available to us. We note that had we included exact information on the timing of the intervention campaign in the catalytic model, our point estimates of incidence of infection before and after the campaign would not likely have changed, although the credible intervals around those point estimates may have been narrower. We present our method for making such estimates without perfect knowledge of the history of control activities because we believe they may prove useful for others conducting similar serologic studies on Chagas, or other diseases, when historical information on control activities is incomplete.

The geography and epidemiology of Chagas disease – like that of many parasitic diseases – is changing. Decreased funding and insecticide resistance are endangering gains achieved by the insecticide-based interventions such as the Southern Cone Initiative against *T. infestans* [38]. Waning political interest further complicates implementation of sustained vector control, and economically and politically marginalized populations may suffer disproportionately. Transmission cycles of *T. cruzi* are emerging in peri-urban communities [32], [33], and re-emerging in peri-rural communities like La Joya. Integrated epidemiological, entomological, environmental and historical data are needed to better elucidate past processes driving the changing geography of Chagas disease, and to facilitate control of new cycles of transmission, especially at the complex rural-urban interface.

## Supporting Information

Checklist S1 Strobe Checklist.
(0.09 MB DOC)
